# Tubule-Guided Cell-to-Cell Movement of a Plant Virus Requires Class XI Myosin Motors

**DOI:** 10.1371/journal.ppat.1002327

**Published:** 2011-10-27

**Authors:** Khalid Amari, Alexander Lerich, Corinne Schmitt-Keichinger, Valerian V. Dolja, Christophe Ritzenthaler

**Affiliations:** 1 Institut de Biologie Moléculaire des Plantes du CNRS, Université de Strasbourg, Strasbourg, France; 2 Department of Botany and Plant Pathology and Center for Genome Research and Biocomputing, Oregon State University, Corvallis, Oregon, United States of America; University of Kentucky, United States of America

## Abstract

Cell-to-cell movement of plant viruses occurs *via* plasmodesmata (PD), organelles that evolved to facilitate intercellular communications. Viral movement proteins (MP) modify PD to allow passage of the virus particles or nucleoproteins. This passage occurs via several distinct mechanisms one of which is MP-dependent formation of the tubules that traverse PD and provide a conduit for virion translocation. The MP of tubule-forming viruses including *Grapevine fanleaf virus* (GFLV) recruit the plant PD receptors called Plasmodesmata Located Proteins (PDLP) to mediate tubule assembly and virus movement. Here we show that PDLP1 is transported to PD through a specific route within the secretory pathway in a myosin-dependent manner. This transport relies primarily on the class XI myosins XI-K and XI-2. Inactivation of these myosins using dominant negative inhibition results in mislocalization of PDLP and MP and suppression of GFLV movement. We also found that the proper targeting of specific markers of the Golgi apparatus, the plasma membrane, PD, lipid raft subdomains within the plasma membrane, and the tonoplast was not affected by myosin XI-K inhibition. However, the normal tonoplast dynamics required myosin XI-K activity. These results reveal a new pathway of the myosin-dependent protein trafficking to PD that is hijacked by GFLV to promote tubule-guided transport of this virus between plant cells.

## Author Summary

To establish infection, plant viruses spread cell-to-cell *via* narrow channels in the cell wall, the plasmodesmata (PD). Movement proteins (MP) are virus-encoded proteins essential for virus intercellular transport through PD. Plasmodesmata located plant proteins (PDLPs), are specifically recognised by the MPs of tubule-forming viruses. Here we show that PDLP targeting to PD depends on the molecular motors myosin XI-K and XI-2. Consistently, and in support of a function of PDLP as PD receptor for MP, overexpression of dominant negative myosin mutants inhibits tubule formation by *Grapevine fanleaf virus* (GFLV) MP and dramatically reduces virus movement.

## Introduction

Plant viruses are intracellular parasites that recruit numerous host factors for their replication and movement within plants. Virus cell-to-cell movement involves transport from replication factories to the cell periphery, passage through plasmodesmata (PD) interconnecting adjacent cells, and long-distance transport *via* the phloem vasculature [1]. All plant viruses encode one or more specialized movement proteins (MP) facilitating virus transport. The structurally and mechanistically diverse MP employ at least three different movement strategies. The first movement strategy is represented by *Tobacco mosaic virus* (TMV) MP that directly binds and chaperones viral RNA genome *via* modified PD [2]–[4]. The second movement strategy involves MP that heavily modify PD structure by forming tubules through which the assembled virions traverse PD [5], [6]. The third type of movement strategies is used primarily by the filamentous viruses, which usually require more than one MP and capsid protein for efficient intercellular transport [7]. The longest known filamentous viruses, closteroviruses, have evolved the most complex machinery that includes a virion-associated movement device and a membrane-targeted MP [8].

Although a number of cellular factors that interact with MPs and/or are localized to PD have been identified, their functional relevance in intercellular transport processes remained largely hypothetical [9]. A new family of PD-resident proteins, Plasmodesmata Located Proteins (PDLPs), was recently characterized in *Arabidopsis thaliana* [10]. PDLPs are type-I membrane proteins that traffic along the secretory pathway to reach the plasma membrane (PM) lining the PD interior. We have recently demonstrated functional significance of PDLP isoforms for movement of tubule-forming viruses including *Grapevine fanleaf virus* (GFLV), an RNA nepovirus causing severe grapevine disease [11]. We showed that PDLPs act as receptors required for assembly of the PD-traversing tubules by the GFLV MP 2B. Inactivation of PDLPs resulted in defective tubule formation and GFLV transport. PDLPs appear to represent essential host components for the tubule-forming movement machinery, because the cell-to-cell movement of the evolutionary dissimilar pararetrovirus, *Cauliflower mosaic virus* (CaMV), was also affected by PDLP down-regulation [11].

One of the central problems in virus transport research is the physical nature of virus translocation within and between cells. Two principal possibilities include diffusion through compartmentalized cytosol and/or endomembrane system and active transport involving cytoskeletal motility. A cytoskeleton-dependent transport route was described in several animal virus models [12] including microtubular motor-driven transport of *Human immunodeficiency virus* (HIV) [13] and actin tail-propelled transport of *Vaccinia virus* [14]. The transport mechanisms of plant viruses remain to be a matter of debate, ironically so for the first virus ever discovered, TMV. For the PD targeting of TMV ribonucleoprotein complexes, evidence has been provided for microtubule-dependent [15], [16] and actomyosin-dependent [17], [18] transport, as well as for diffusion in the endoplasmic reticulum (ER) network [19]. Although these mechanisms are not necessarily mutually exclusive, it seems that the growing number of plant viruses are reported to recruit actomyosin for moving their genomes, virions, or MPs to or through PD [20].

The actomyosin motility in plants, from algae to angiosperms, is driven by two classes of myosin motors, VIII and XI, which are evolutionary related to class V myosins present in protists, fungi, and animals [21]. The model plant *Arabidopsis thaliana* encodes 13 class XI and four class VIII myosins [22]. Class XI myosins function in the trafficking of Golgi stacks, peroxisomes, mitochondria, and ER streaming [23]–[25]. Because inactivation of Arabidopsis class XI myosins affects cell growth and plant development [26], [27], these molecular motors are likely to transport the secretory vesicles required for cell expansion. Although myosins VIII were proposed to associate with PD, ER, plasma membrane, and endosomes [28]–[30], in the absence of genetic evidence, their functional significance remains a mystery.

The first experimental support for actomyosin-dependent PD targeting of a viral protein was provided for a closteroviral Hsp70 (Heat shock protein 70) homolog, a virion component required for viral movement [31], [32]. It was also shown that Hsp70 localization to PD specifically relies on class VIII myosins [33]. Very recently, it was found that MP of a dissimilar tenuivirus also relies on myosins VIII for PD targeting [34]. In contrast, myosins XI were recently implicated in TMV movement [18].

In this study, we investigate the role of the actomyosin motility in PD-targeting of PDLP, and consequently, in tubule-guided cell-to-cell movement of GFLV. We demonstrate that myosins XI, but not VIII, mediate intracellular trafficking and PD targeting of the GFLV MP receptor PDLP. We show that inactivation of certain class XI myosins affects GFLV cell-to-cell movement. Furthermore, we explore the roles of myosins XI in the subcellular targeting of several compartment-specific fluorescent reporters. Taken together, our data delineate a specific, myosin XI-dependent, endomembrane transport pathway for PD-localised plant proteins that contributes to GFLV transport between the cells.

## Results

### GFLV cell-to-cell movement is actomyosin-dependent

**Figure 1:**
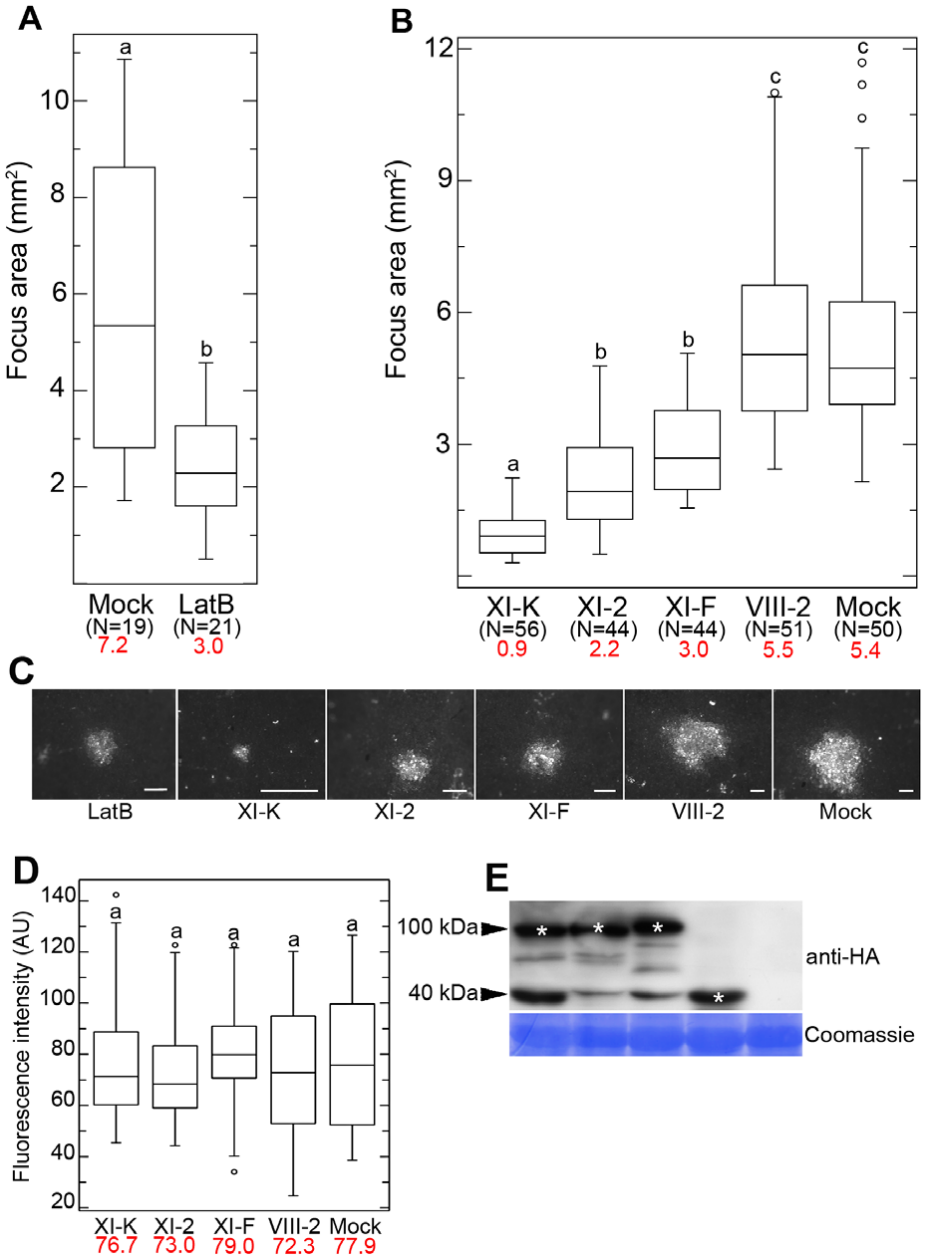
Effects of LatB treatment and myosin tail expression on cell-to-cell movement of GFLV. (A) Mean size of GFLV-RFP infection foci at 3 dpi is reduced in 10 µM LatB treated leaves compared to control leaves. (B) Transient expression of the myosin XI-K tail strongly inhibits GFLV-RFP cell-to-cell movement with the myoisn XI-2 and XI-F tails having significant, but less strong effects, and myosin VIII-2 tail being indistinguishable from the mock-infiltrated control. (C) Representative images of the GFLV-RFP infection foci formed in the leaves expressing myosin tails as indicated. Scale bars, 1 mm. (D) Fluorescence intensity (AU, arbitrary units) of the same foci shown in (B) was not significantly different among all experimental conditions. (E) Immunoblot analysis using HA-specific antibodies showed similar expression levels for the HA-tagged myosin tails in the inoculated leaves used in (B) and (C). Bands corresponding to the class XI (approximately 100 kDa) and class VIII (approximately 40 kDa) myosin tails are marked by asterisks. Coomassie blue staining (bottom panel) shows equal loading. Each box plot depicts measurements from the 25th to 75th percentile. The error bars correspond to the 10th and 90th percentiles. The horizontal bar in each box represents the median value. The circles indicate outliers. Different letters (a, b and c) above the box plots indicate statistically significant differences between the different treatments determined by *t*-test (P<0.001) (A) and ANOVA (P<0.05) (B and D). N, total number of the foci analysed in 3 independent experiments. Mean values are given in red.

To determine if a functional actin cytoskeleton is required for cell-to-cell movement of GFLV, we applied the actin microfilament depolymerising agent Latrunculin B (LatB) [35] to *Nicotiana benthamiana* leaves before infection. GFLV cell-to-cell movement was assessed 3 days post inoculation (dpi) by measuring the size of infection foci of a recombinant GFLV encoding red fluorescent protein-fused reporter (GFLV-RFP) [11]. Box plot was used as statistical method to study the range of infection foci diameters in the different treatments. Figure 1A shows a ∼2.5-fold reduction in mean infection focus area in the LatB treated leaves compared to the control, indicating that GFLV spread requires an intact actomyosin motility system.

Because the GFLV MP or small icosahedral virions were unlikely to induce actin tail formation similar to large poxviruses [14], we assumed that the myosin motors were involved in virus intercellular movement. To address this possibility, we used dominant negative inhibition of myosin function *via* transient overexpression of the headless myosins that possess C-terminal globular tail domains. Because these domains are specifically involved in myosin cargo binding and motor domain activation [36], [37], their ectopic expression suppresses activity of the endogenous myosins. This approach was successfully used for the interference with the functions of myosins VIII and XI in *N. benthamiana* and Arabidopsis [23], [24], [33]. It is important that we expressed *N. benthamiana* myosin tails in this same plant species, because heterologous myosin expression often results in mislocalization (Peremyslov VV and VV Dolja, unpublished data).

The myosin tail-expressing and control leaves were inoculated with GFLV-RFP, and the resulting infection foci were measured at 3 dpi. We found that the inhibition of the myosins XI affected the size of the infection foci (Figures 1B and C). The boxes for all myosin XI tails treatments are compressed in comparison with VIII-2 and control (Figure 1B) indicating less distribution around the median showing lower median. These results indicate an affect of the expression of myosin XI tails on virus cell-to-cell movement. Expression of the myosin XI-K tail had the most dramatic effect reducing virus cell-to-cell movement by factor 6 compared to the control. Similar, albeit milder effects were observed upon expression of the myosin XI-2 and XI-F tails (Figures 1B and C). By contrast, virus movement was not significantly different from the control when myosin VIII-2 tails were expressed (Figures 1B and C). The expression of the hemagglutinin epitope (HA)-tagged myosin VIII-2, XI-K, XI-2, and XI-F tails [23] was detected using immunoblot analysis and anti-HA antibodies. This analysis confirmed that the recombinant proteins had the expected sizes (myosins VIII possess much shorter tails than those of myosins XI) and similar levels of accumulation (Figure 1E).

The reduction in the size of infection foci could be attributed either to a defect in virus transport between cells, or to a reduction in virus replication in response to myosin tail expression. To address the latter possibility, we quantified GFLV-RFP fluorescence intensity normalised to the area in a large number of infection foci (N ≥44 for each experimental variant). This analysis unequivocally demonstrated that there were no significant differences between the control and each of the myosin tail-expressing variants (Figure 1D). We therefore concluded that the dominant negative inhibition of the myosin XI-K, and to a lesser extent, of myosins XI-2 and XI-F, specifically affected the cell-to-cell movement of GFLV.

### Class XI myosins facilitate tubule formation in virus-infected cells

Because GFLV cell-to-cell movement occurs *via* tubules assembled by 2B MP [11], [38], we were interested to determine if tubule formation is impaired upon myosin XI tail expression. To this end, we used transient co-expression of the myosin tails and the GFP:2B that is able to form tubules [38] and assessed tubule formation using confocal laser scanning microscopy.

**Figure 2:**
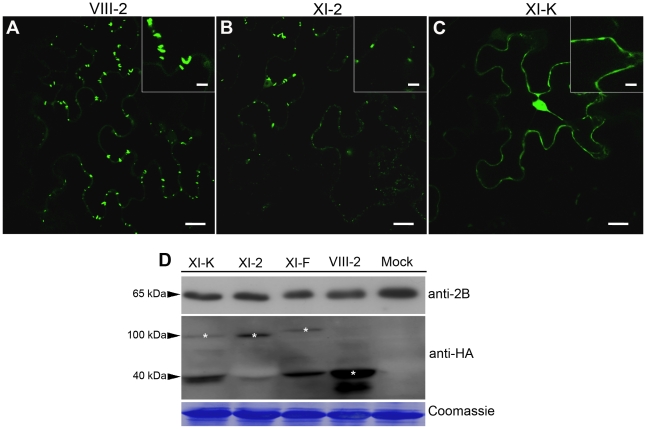
Transient expression of the myosin XI-K tail disrupts formation of the PD-associated tubules by the GFLV movement protein GFP:2B. (A) Representative confocal image showing normal, PD-localized tubules formed by GFP:2B in control leaves co-infiltrated with empty vector-transformed Agrobacterium, myosin VIII-2 or XI-F tails. (B) Co-expression of the myosin XI-2 tail reduces tubules formation by GFP:2B. (C) Co-expression of the myosin XI-K tail results in the nucleo-cytoplasmic redistribution of GFP:2B; no tubules are formed under these conditions. Insets are shown in higher magnification. (D) Immunoblot analysis using 2B- (top panel) or HA-specific (middle panel) antibodies revealed similar expression levels for GFP:2B, as well as somewhat variable levels of the HA-tagged myosin tails. Bands corresponding to the class XI (100 kDa) and class VIII (40 kDa) myosin tails are marked by asterisks. Coomassie blue staining (bottom panel) shows equal loading. Scale bars, 20 µm; inside insets, 10 µm.

As expected, no effect on tubule assembly was observed when GFP:2B was transiently co-expressed with the tail of myosin VIII-2 (Figure 2A). Ectopic expression of the myosin XI-2 tail resulted in fewer as well as shorter tubules (Figure 2B, Compare insets in figure 2A and B). The most conspicuous effect on tubule formation was observed upon expression of the myosin XI-K tail. As shown in Figure 2C, no discernible tubules were observed in this case. Instead, GFP:2B was distributed diffusely in the cortical cytoplasm and nucleus, attesting to a major disruption of not only the tubule assembly, but also GFP:2B localization at PD (Figure 2C). Immunoblot analysis using 2B- and HA-specific antibodies confirmed co-expression of GFP:2B and each of the myosin tails (Figure 2D). These data clearly indicated that functional myosins XI in general, and myosin XI-K in particular, are required for proper subcellular targeting of the GFLV MP, and subsequent formation of the PD-traversing tubules by this protein.

### PDLP1 trafficking is driven by the actomyosin motility system

**Figure 3:**
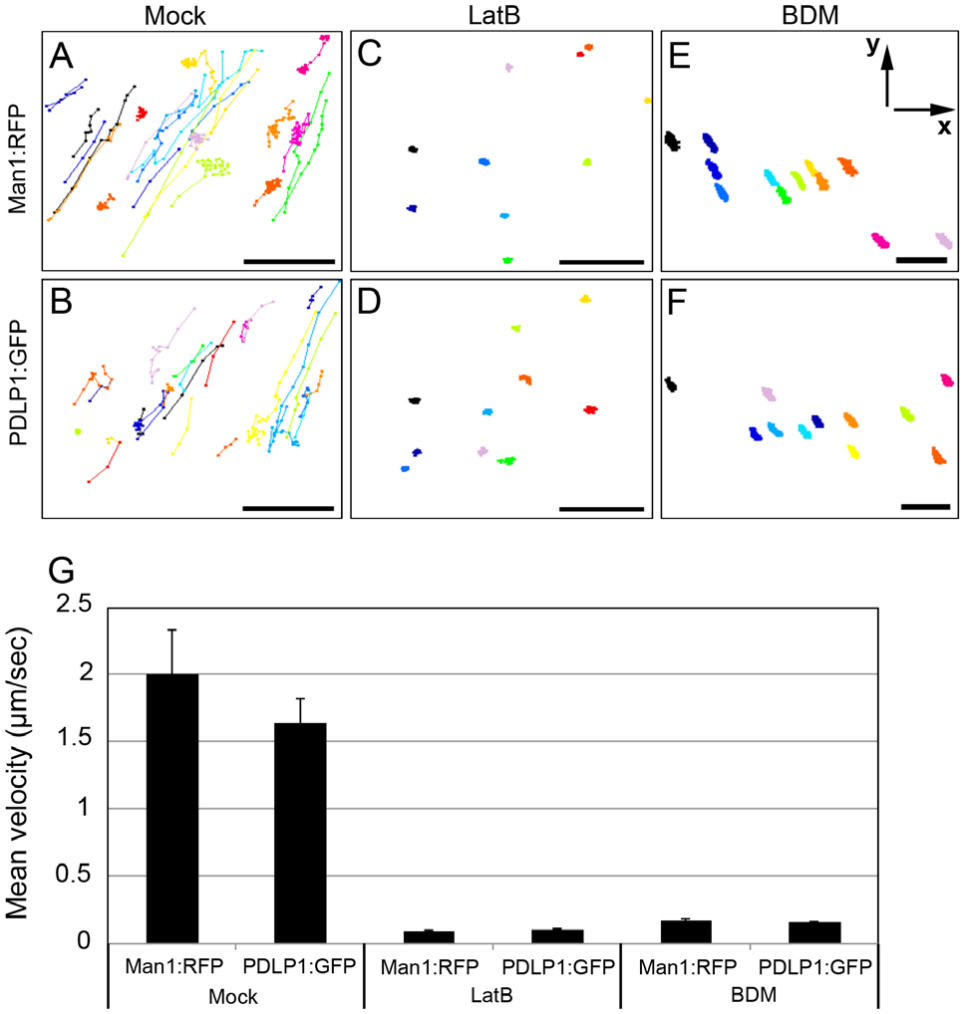
Effects of the microfilament inhibitor LatB and myosin inhibitor BDM on trafficking velocity of Golgi stacks marked by Man1:RFP or PDLP1:GFP labelled bodies. (A–F) Tracks, represented by different colours, of the individual Man1:RFP fluorescent Golgi stacks (A, C, E) or PDLP1:GFP-tagged bodies (B, D, F) were imaged for 2 min in the presence of 0.1% DMSO (Mock; A, B), 10 µM LatB (C, D), or 10 mM BDM (E, F) and depicted *via* connected dots. (G) Mean of Man1:RFP (Golgi marker) and PDLP1:GFP velocity were calculated using images used in A–F. Number of investigated Golgi stacks and PDLP1:GFP, ≥27 (mock); ≥10 (Lat B, BDM). Axes are given in panel E. Scale bars, 10 µm.

We demonstrated previously that accumulation of PDLP isoforms at PD is crucial for tubule formation by GFLV MP and virus cell-to-cell movement [11]. To determine if PDLP trafficking along the ER-to-Golgi-to-PD pathway [10] requires actomyosin motility, we co-expressed GFP-tagged PDLP1 (PDLP1:GFP) and the spectrally distinct Golgi marker Man1:RFP [39]. Man1:RFP served as an internal control for Golgi motility in experiments using LatB to test whether PDLP1 movement is actomyosin-dependent. Additionally, the ATPase inhibitor 2,3 butanedione monoxime (BDM) reported to inhibit myosin activity [39], [40] was applied to assess the role of myosins in PDLP1 trafficking to PD. As shown in Figure 3, translocation of Man1:RFP (Figure 3A) and PDLP1:GFP-labelled bodies (Figures 3B and video S1) under control conditions occurs along considerably overlapping tracks (compare Figures 3A and B) and with similar velocities of 1.64 µm/sec ± 0.18 and 2.01 µm/sec ± 0.32, respectively (Figure 3G). Strikingly, LatB treatment nearly abolished trafficking of both Man1:RFP (Figure 3C) and PDLP1:GFP (Figure 3D). The resulting measured mean velocities of less than 0.11 µm/sec (Figure 3G) are attributed most likely to Brownian motion-dependent wobbling, cytosol dynamics due to the activity of microtubule-associated motors, and/or the drift of the entire specimen. Very similar results were obtained upon BDM treatment (Figures 3E and G). Statistical analyses revealed highly significant velocity differences between control (mock) and LatB or BDM treatments (*t*-test, *p*<0.01, Figure 3G). Taken together, these results suggest that trafficking of PDLP1 bodies occurs *via* a route similar to that of Man1:RFP-labelled Golgi stacks, and is actomyosin-dependent.

### PDLP1 localization to PD specifically requires class XI myosins

**Figure 4:**
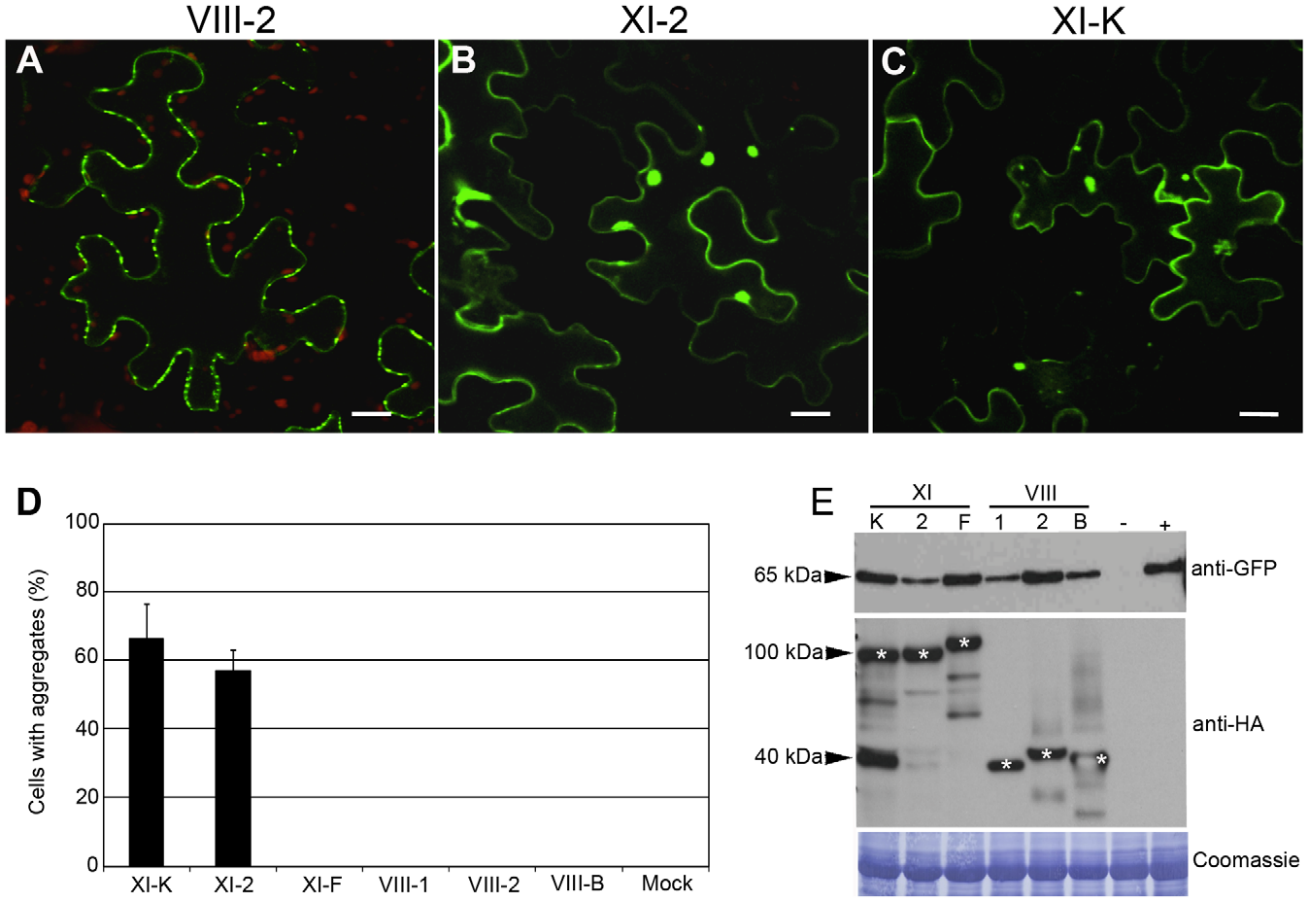
Transient co-expression of PDLP1:GFP and myosin XI-K or XI-2 tails leads to the PDLP1:GFP mislocalization and aggregation. (A) PDLP1:GFP co-expressed with empty vector displays normal PD localization. Similar localization is observed in the presence of the myosin VIII-1, VIII-2, VIII-B, or XI-F tails (not shown). (B and C) Co-expression of PDLP1:GFP with the myosin XI-2 tail (B) or myosin XI-K tail (C) results in the distribution of PDLP1:GFP to the cell periphery and to formation of large cytosolic aggregates. Scale bars, 20 µm. (D) Percentage of cells showing PDLP1:GFP aggregates upon co-expression with the different myosin tail variants. Error bars indicate standard error of the mean of 3 independent experiments; between 200 and 300 cells were analysed for all experimental variant. (E) Immunoblot analysis of leaves infiltrated with PDLP1:GFP (approximately 65 kDa; top panel) and myosin tails (middle panel; marked with asterisks) as indicated. Mock-infiltrated and PDLP1:GFP controls are marked (−) and (+), respectively. The coomassie-blue stained PVDF membrane to validate equal loading is shown in bottom panel.

To investigate the potential myosin contributions to PDLP1 transport to PD, we co-expressed PDLP1:GFP with the tails of class VIII and XI myosins including VIII-1, VIII-2, VIII-B, XI-K, XI-2, and XI-F. Figure 4A to C shows representative images of this analysis. The normal pattern of PDLP1:GFP localization to PD was observed in empty vector control and in the leaves expressing each of the three class VIII myosin tails (Figure 4A), or the myosin XI-F tail (not shown). In a sharp contrast, expression of the myosin XI-2 (Figure 4B) or myosin XI-K (Figure 4C) tails resulted not only in disruption of the specific PD targeting as seen by the absence of typical punctate labelling in the cell wall, but also in formation of multiple abnormal PDLP1:GFP aggregates in the cytosol (Figures 4B and C). Three independent experiments revealed that approximately 60% of the epidermal cells expressing myosin XI-2 or XI-K tails presented such aggregates, whereas no PDLP1 aggregates were detected in any other experimental variants (Figure 4D).

To determine if PDLP1:GFP mislocalization and/or aggregation was due to excessive protein accumulation, we analyzed the steady-state levels of PDLP1:GFP and myosin tails using immunoblotting and GFP- or HA-specific antibodies, respectively. As expected, GFP-specific antibodies revealed a 62-kDa PDLP1:GFP-specific product in all samples except for the mock-infiltrated control [lane (-) in Figure 4E, top panel]. Although protein levels varied slightly between the different experimental conditions, no correlation between high levels of PDLP1:GFP expression and aggregate formation was observed. Indeed, highest PDLP1:GFP accumulation levels were seen with samples expressing myosin XI-F and VIII-2 tails (Figure 4E), where no aggregates were formed (Figure 4D). Conversely, samples expressing myosin XI-2 tails exhibited the lowest PDLP1:GFP accumulation, and nearly 60% of the corresponding cells showed PDLP1:GFP aggregates (Figures 4D and E). In regard to the myosin tail expression, accumulation levels were very similar both for class XI myosin tails (approximately 100 kDa) and VIII (approximately 40 kDa) (Figure 4E, asterisks). We concluded that, among the 6 tested myosins, only expression of the myosin XI-2 and XI-K tails specifically induced mislocalization and abolished PD targeting of PDLP1:GFP.

**Figure 5:**
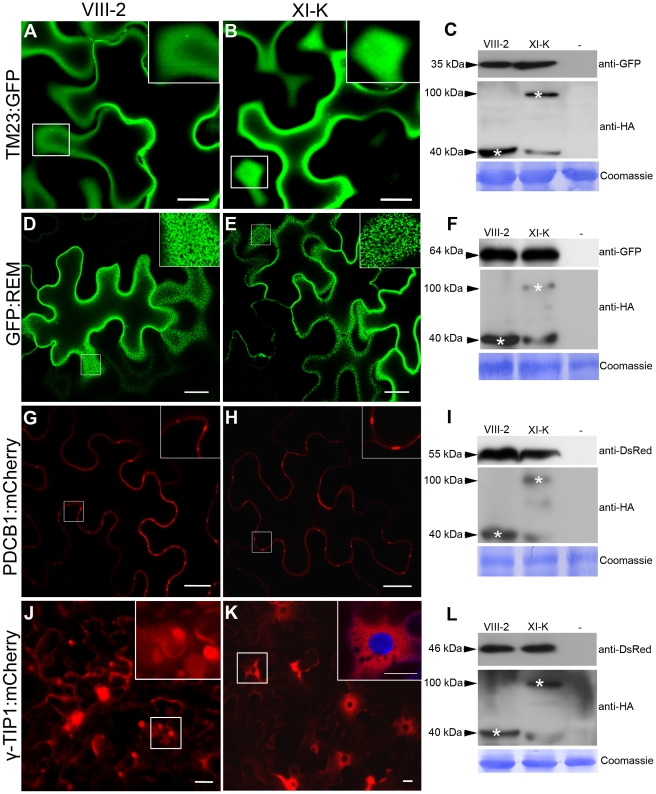
Effects of myosin XI-K tail expression on the localization of different cellular markers in *N. benthamiana*. (A–B) The subcellular localization of the TM23:GFP marker at the PM is not affected by expression of myosin XI-K tails. (D–E) No effect on the lipid raft/PM localization of GFP:REM was observed upon coexpression with myosin XI-K tails. (G–H) The normal localization to PD necks of the PDCB1:mCherry was not affected by expression of myosin XI-K tails. (J–K) Co-expression of the myosin XI-K tail (K) with the tonoplast marker γ-TIP1:mCherry inhibits formation of the tonoplast-derived bulbs (J, inset) and leads to uniform distribution of the marker within the tonoplast and in the perinuclear compartment (K, inset. DAPI staining shows nucleus). Scale bars, 20 µm. (C, F, I, L) The expression of the cellular markers (top panels) and myosin tails (middle panels) in each experimental variant was validated using immunoblot analysis and antibodies as indicated. Bottom panels show equal loading verified by Coomassie-staining.

The myosins XI-K and XI-2 are the principal drivers of cell dynamics including organelle trafficking and F-actin organization, as well as diffuse and polarized cell growth [25]–[27]. Therefore, we were interested to determine if the contributions of these same myosins to PDLP1 localization were general or affected a specific targeting pathway. Firstly, we addressed a potential role of myosins in protein targeting to the plasma membrane (PM) using the PM marker TM23:GFP [41]. As shown in Figure 5A and 5B, the distribution pattern of TM23:GFP was not affected by the expression of myosin VIII-2 or XI-K tails; in both cases the marker was localized throughout the PM. Co-expression of the marker and myosin tails in all experimental conditions were validated by immunoblot analysis (Figure 5C).

Secondly, we investigated localization of the green fluorescent protein-tagged remorin (GFP:REM), a membrane microdomain marker localized in the PM and in PD [42]. Contrarily to PDLP1, remorin down regulate virus cell-to-cell movement by interacting directly with, MP of a potexvirus. As described [42], GFP:REM clustered in discrete PM domains; this localization pattern was not altered upon the transient expression of myosin tails VIII-2 (Figure 5D) or XI-K (Figure 5E). Immunoblot analysis confirmed expression of GFP:REM and myosin tails in each experimental variant (Figure 5F).

Thirdly, we examined the targeting of the plasmodesmata callose binding 1 (PDCB1) protein fused to mCherry (PDCB1:mCherry), which is localized to the PD neck region [43]. Once again, overexpression of the tails of myosin VIII-2 (Figure 5G) or XI-K (Figure 5H) had no observable effect on PDCB1:mCherry targeting to PD-enriched areas at the cell periphery (see Figure 5I for the PDCB1:mCherry and myosin tail expression). Collectively, these results show that in contrast to PDLP1:GFP or GFP:2B the transport or retention of the three tested protein markers targeted to the PM and/or PD was not affected by overexpression of the myosin VIII-2 or XI-K tails, indicating a distinct PD-transport route for PDLP1:GFP.

Finally, we were interested to determine if the transport along the secretory pathway directed to the vacuole membrane (tonoplast) rather than to the PM is myosin-dependent. This question was addressed using the tonoplast-specific marker γ-TIP1 fused to mCherry (γ-TIP1:mCherry; [44]). The vacuoles in fully expanded plant cells usually account for the most of cell volume [45]. The tonoplast surrounding these gigantic organelles is constantly reshaped *via* formation of the transvacuolar strands and spherical tonoplast invaginations often called bulbs [46]. The tonoplast and bulbs were readily visualized in the γ-TIP1:mCherry-expressing control cells, as well as in myosin VIII-2-expressing cells (Figure 5J). Interestingly, expression of the myosin XI-K tails abolished the bulb formation and led to enrichment of γ-TIP1:mCherry in the perinuclear tonoplast domain (Figure 5K). As in the previous experiments, similar levels of the marker and myosin tail accumulation were confirmed using immunoblot analysis (Figure 5L). We concluded that although the transport of tonoplast-targeted protein was unaffected upon myosin tail expression, the normal tonoplast dynamics required myosin XI-K activity. In general, our observations using dominant negative expression of myosin tails suggest that specific inhibition of myosin XI-K activity affected a specific trafficking pathway of PDLP1 to PD rather than caused an indiscriminate suppression of the endomembrane transport.

## Discussion

The role of cytoskeletal motility in viral infection is a rapidly progressing albeit relatively young field of research at the frontiers of virology and cell biology. The microtubule-dependent transport of retroviruses to nucleus [47] and Herpesvirus to axon endings [48], actin-dependent formation of the virological synapses through which HIV moves between cells [49], and an actin-tail propelled transport of poxviruses [50] are a few illuminating discoveries in this field. Animal and plant viruses share multiple replication mechanisms that rely on conserved features of eukaryotic cells [51], [52]. In contrast, virus cell-to-cell movement in plants occurs *via* the plant-specific PD, channel-like organelles providing symplasitc continuity between adjacent cells [53]. To accomplish movement through PD, plant viruses have evolved dedicated MPs that target and modify PD to mediate virus passage. One of the principal mechanisms of MP action is a tubule-guided PD transport used by a wide variety of the RNA and retroid DNA viruses [1] whereby MP modifies PD by assembly into multimeric tubules through which virion movement occurs.

Most of the previous work on plant virus-cytoskeleton relationships involved chemical inhibitors [20]. Although useful for an initial insight, this approach is not unlike a sledgehammer because global disruption of microtubules or microfilaments causes dramatic changes in cell physiology that are difficult to associate with specific mechanisms of virus replication or transport. Even in the cases like TMV, where genetic and other more subtle approaches were used [16], [18], [19], [54], the picture is less than clear. In a large part, difficulties in reconciling work from different labs stem from the incomplete understanding of the cellular partners required for the MP function. Our recent discovery of PDLPs as host receptors [10], [11] that mediate PD targeting of the tubule-forming MPs of the nepovirus GFLV and the caulimovirus CaMV provided a unique opportunity to address the role of actomyosin motility in virus transport using both the chemical and the more specific dominant negative inhibition of myosins [23], [33].

Combining these approaches, we revealed critical contributions of the myosin motors in the GFLV transport between the cells. We identified myosin XI-K as a principal driver of this process with additional contributions provided by other class XI, but not class VIII myosins. Furthermore, we obtained important new insight into myosin-driven endomembrane transport in plants by showing that myosin XI-K acts in a specific pathway within a general ER-to-Golgi-to-PM transport network.

Because GFLV transport is tubule-dependent, it was important to determine if myosin inactivation interfered with tubule formation or PD localization. Our previous work using suspension cell culture has shown that tubule assembly requires ER-to-Golgi pathway, whereas cytoskeletal systems appeared to contribute to tubule targeting [38]. Here, we found that the inhibition of myosin XI-K resulted in a conspicuous nucleo-cytosolic redistribution of the GFP:2B with no detectable PD-associated tubules. Thus, tubule formation was specifically affected by myosin inactivation.

As was demonstrated recently, 2B assembles tubules at PD *via* interaction with the host PDLP receptors [11] that, in turn, are transported to PD along the ER-to-Golgi pathway [10]. Therefore, both GFLV movement and tubule formation at PD require proper PDLP targeting. To determine if PDLP targeting was actomyosin dependent, we investigated PDLP1:GFP transport pathway using cytoskeletal inhibitors and dominant negative inhibition of the individual myosins. We found that PDLP1:GFP was present in mobile bodies whose rapid trafficking was abolished by application of LatB or BDM similarly to Golgi stacks whose transport in plants relies entirely on myosins XI [27].

Furthermore, we showed that the myosins XI-K and XI-2, but not XI-F, VIII-1, VIII-2, and VIII-B are required for PDLP1 delivery to PD. Inactivation of the two former myosins resulted in PDLP1:GFP redistribution in the cortical cytoplasm and inclusion bodies that were never observed in the cells where other myosins were inhibited. Given the strong correlation between disruption of PDLP targeting and GFLV movement by interference with myosins XI-K and XI-2 (Figures 1B and 4B-D), we propose that the primary contribution of these myosins to virus transport is the delivery of PDLP-receptors to PD. It is important to stress that this result is also the first indication of myosin XI function in the trafficking of secretory vesicles to the PM/PD compartment.

The next question to ask was if PDLP transport occurred along a common post-Golgi secretory pathway, or represented a specialized route within this pathway driven primarily by myosins XI-K and XI-2. To address this question, we assessed a role of myosin XI-K in the targeting of markers differentially localized to: i) entire PM; ii) lipid raft subdomains within PM and PD; iii) PD neck or iv) vacuolar membrane (tonoplast). We found that proper targeting of the former three markers was not affected by myosin XI-K inhibition suggesting that the myosin XI-K-dependent PDLP targeting represents a specific route within a broad endomembrane transport network. In addition, we found that myosin XI-K is required for the normal tonoplast reshaping *via* transient invaginations.

It was previously demonstrated that PD targeting of the closteroviral Hsp70 homolog requires myosins VIII [33], although significance of this process for virus movement was not addressed. It was also found that myosin XI-2 knockdown reduced TMV movement [18], but this effect was not linked to a specific mechanism. Together with our previous work [10], [11], [38], this study provides a basis for an advanced mechanistic model of myosin-dependent virus movement.

**Figure 6:**
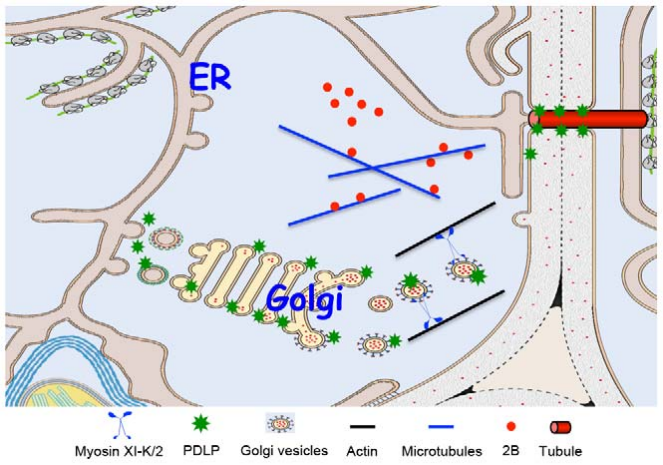
Model for PDLP and GFLV MP 2B targeting to PD and tubule formation. The GFLV MP (2B) reaches PD by diffusion or by microtubule – mediated transport. PDLP traffics along the secretory pathway and its post-Golgi delivery to the plasmamembrane and/or PD relies on myosin XI-K and myosin XI-2. Within PD, interaction between 2B and PDLP promotes tubule formation to allow GFLV virion cell-to-cell movement.

According to this model (Figure 6), the GFLV MP and its host receptor, PDLP, traffic to the cell periphery along distinct pathways. 2B reaches PD by diffusion or by association with microtubules [38]. The transport route employed by PDLP is dependent on the myosins XI with XI-K playing the principal role. At PD, MP binds PDLP for anchorage and tubule assembly. Because transient inhibition of PDLP traffic to PD reduces virus movement (Figure 1), it seems that steady-state supply of this receptor is required for the formation of tubules that restructure PD. Finally, assembled GFLV virions enter tubules and translocate into adjacent cells. It remains to be determined if virion transport to and through tubules involves cytoskeleton-dependent motility.

The emerging picture of the plant-virus interactions with myosin motors is complex and nuanced. It appears that closteroviral Hsp70 homolog directly recruits myosins VIII for virion delivery to PD [33], whereas tenuiviral MP uses myosin VIII-assisted vesicular transport for the same task [34]. Currently, the PD-directed transport of these viral proteins remains the only experimentally supported function of the class VIII myosins. On the other hand, TMV MP targeting to PD does not require myosins [34], whereas myosin XI-2 facilitates TMV movement likely *via* delivering the ER-associated viral replication complexes to PD [18], [20], [55], [56]. This latter hypothesis resonates well with the role of myosins XI-2 and XI-K in ER transport [25]. In the case of GFLV presented here, the virus relies on the myosins XI-K and XI-2 for the trafficking of the host MP receptor PDLP to PD.

In addition to important insight into virus-cytoskeleton interactions, our work suggests novel functions of the myosins XI-K and XI-2 in vesicle trafficking and vacuole remodelling. These myosins were previously shown to drive the trafficking of Golgi stacks, peroxisomes, and mitochondria [26], [27], as well as the ER flow [25]. Here we show that these same myosins are also involved in PDLP delivery to PD *via* a specific endomembrane transport pathway, as well as in remodelling of the vacuolar membrane. Further inquiries into the mechanisms of myosin-dependent transport are certain to deepen our understanding of the cell interior dynamics and the importance of these processes for virus movement.

## Materials and Methods

### Plant material and virus inoculation

All experiments were performed using *N. benthamiana*, an experimental GFLV host that supports the complete systemic infection cycle. The plants were grown in growth chambers under 16/8h light/dark cycles, 24/20°C day/night temperatures and approximately 70% humidity. Agroinfiltrated and/or virus-infected leaves were of the same age and size and were maintained at the same conditions. Approximately 300 ng of purified GFLV-RFP virions was mechanically inoculated into *N. benthamiana* leaves.

### Transient protein expression

The binary vectors designed to express HA-epitope tagged *N. benthamiana* myosin tails VIII-1, VIII-2, VIII-B, XI-K, XI-F, and XI-2 were described earlier [33]. The fluorescent reporter proteins used to visualize subcellular compartments were as follows: GFP:2B, the GFLV MP forming tubules at PD of virus-infected cells [11]; PDLP1:GFP localized in the PM lining PD channel [10]; PDCB1:mCherry targeted to the PD neck [43]; GFP:REM localized to lipid rafts within PM and PD [42]; TM23:GFP labelling the entire PM [41]; Man1:RFP associated with Golgi-stacks [39]; tonoplast-specific γ-TIP1:mCherry [44]. All plasmids were transformed into *Agrobacterium tumefaciens* (strain LBA4404) that was used for agroinfiltration at a final optical density (OD 600 nm) of 0.3 [11]. Leaf samples were processed for imaging or immunoblot analysis at 48 hours post infiltration.

### Drug treatments

To analyze the effect of the actin microfilament disassembly drug LatB on GFLV infection, *N. benthamiana* leaves were infiltrated with 10 µM LatB in 0.1% DMSO 6 hours prior to inoculation with GFLV-RFP. In addition, 10 µM LatB or 10 mM 2,3 butanedione monoxime in water solution (BDM; an ATPase inhibitor that disrupts myosin function) were vacuum infiltrated into *N. benthamiana* leaf disks 36 hours after agroinfiltration to examine the effects of these inhibitors on the trafficking and localization of PDLP1:GFP and Man1:RFP. Leaf disks were kept in a moisture chamber and were observed at 12 hours after the treatment. Control infiltrations were performed either with 0.1% DMSO or with water.

### Immunoblot analysis

Total protein extracts were obtained by grinding *N. benthamiana* leaf disks in Laemmli buffer, separated by SDS-PAGE, and transferred by electroblotting to a polyvinylidene difluoride membrane (Immobilon-P; Millipore). To detect myosin tails, membranes were probed with anti-HA-peroxidase antibodies (Sigma-Aldrich) at 1∶5,000 dilution. For the GFLV movement protein 2B, affinity purified GFLV 2B-specific rabbit antibody [57] was used in 1∶10,000 dilution. The expression of all other GFP-tagged proteins was assayed using, the monoclonal anti-GFP antibodies (Clontech) diluted to 1∶5,000. The expression of mCherry-fused γ-TIP1 and PDCB1 was detected using polyclonal anti-DsRed antibodies as recommended by manufacturer (Clontech).

### Confocal laser scanning microscopy and image processing

Cells expressing fluorescent proteins were imaged using a Zeiss LSM510 laser scanning confocal microscope with a C-Apo-chromat (63X/1.2 W Korr) water objective lens under multitrack mode. Excitation/emission wavelengths were 488 nm/505 to 545 nm for GFP and 543/long pass 560 nm for RFP. Confocal images were processed using LSM510 software version 2.8 (Zeiss).

GFLV-RFP infection foci were examined under a Leica MacroFluo epifluorescent microscope equipped with the apochromatically corrected zoom system Z16 APO, a 5x objective and a DFC 360FX camera. All imaging was conducted under identical illumination and exposure conditions to allow comparisons. Following acquisition, images were processed using ImageJ (1.38u), and Adobe Photoshop (v7.0) software.

### Statistical analyses

Statistical evaluations were made using ANOVA R software or Student's *t*-test where appropriate.

## Supporting Information

Video S1 PDLP1:GFP overexpressed in *N. benthamiana*. PDLP1:GFP-labelled bodies were observed 48 hours after agroinfiltration.
(MOV)
